# Unplanned Excision of Synovial Sarcoma: Factors Associated with Recurrence and Survival

**DOI:** 10.3390/cancers16183157

**Published:** 2024-09-14

**Authors:** Samuel E. Broida, Alexandra M. Arguello, Mikaela H. Sullivan, Steven I. Robinson, Scott H. Okuno, Brittany L. Siontis, Thanh P. Ho, Peter S. Rose, Meng Xu-Welliver, Matthew T. Houdek

**Affiliations:** 1 Department of Orthopedic Surgery, Mayo Clinic, Rochester, MN 55905, USA; broida.samuel@mayo.edu (S.E.B.); arguello.alexandra@mayo.edu (A.M.A.); sullivan.mikaela2@mayo.edu (M.H.S.); rose.peter@mayo.edu (P.S.R.); 2 Department of Medical Oncology, Mayo Clinic, Rochester, MN 55905, USA; robinson.steven@mayo.edu (S.I.R.); okuno.scott@mayo.edu (S.H.O.); siontis.brittany@mayo.edu (B.L.S.); ho.thanh@mayo.edu (T.P.H.); 3 Department of Radiation Oncology, Mayo Clinic, Rochester, MN 55905, USA; welliver.meng@mayo.edu

**Keywords:** synovial sarcoma, unplanned excision, soft tissue sarcoma

## Abstract

**Simple Summary:**

Unplanned excision of soft tissue sarcoma presents a challenge for treating physicians, both in terms of prognostication and management. We retrospectively reviewed the outcomes and factors associated with recurrence and survival among patients with synovial sarcoma who were diagnosed after unplanned excision. We found that synovial sarcoma is initially excised in a non-oncologic fashion at a high rate, and those who had residual tumor on re-excision fared worse than those without residual tumor. We suggest that margin-negative re-resection and radiotherapy be strongly considered for those who previously have undergone unplanned excision of synovial sarcoma.

**Abstract:**

**Background**: Synovial sarcoma is rare and may present as a small, slow-growing mass. These tumors are often mistaken as benign and are therefore prone to unplanned and/or non-oncologic excision. We sought to identify the rate of unplanned excision of synovial sarcoma and risk factors for recurrence and survival among this cohort. **Methods**: The medical records of 246 patients evaluated at a single institution for synovial sarcoma between 1997 and 2022 were retrospectively reviewed. Of these, 87 (35%) underwent unplanned, non-oncologic excision. The mean age of the cohort was 49 years. Primary tumors were located in the extremity (n = 63), abdomen (n = 6), thorax (n = 7), head/neck (n = 8), and paraspinal region (n = 3). The median maximum pre-treatment dimension of the primary tumor was 4.8 cm (IQR 7–2.4). Seventy-seven (86%) patients underwent re-excision of the tumor bed, 39 (45%) received chemotherapy, and 63 (72%) received radiation therapy. **Results**: Among patients who underwent unplanned excision, local recurrence-free survival (LRFS) was 98% at 1 year and 82% at 5 years. Metastasis-free survival (MFS) was 91% at 1 year and 72% at 5 years. Disease-specific survival (DSS) was 98% at 1 year and 72% at 5 years. When adjusting for tumor size, tumors which underwent unplanned excision did not have worse recurrence or survival compared to those which had planned excision (*p* > 0.10). Size > 5 cm, monophasic subtype, and axial location were associated with increased risk of disease recurrence. Forty-six patients had residual tumor following re-excision, which was associated with worse MFS (HR 8.17, 95% CI [1.89, 35.2], *p* < 0.01) and DSS (HR 7.66, 95% CI [1.76, 33.4], *p* < 0.01). Patients who received radiotherapy had improved MFS (HR 6.4, 95% CI [1.42, 29.0], *p* = 0.02) and DSS (HR 5.86, 95% CI [1.27, 26.9], *p* = 0.02). **Conclusions**: One-third of patients presenting with synovial sarcoma were diagnosed after unplanned, non-oncologic excision. Patients with large, axial tumors had worse survival. Approximately half of patients who underwent unplanned excision had no residual tumor after pre-operative radiation. The use of radiation was associated with decreased rates of recurrence and improved disease-specific survival. Our results suggest that margin-negative re-resection and radiotherapy should be considered when feasible following unplanned excision of synovial sarcoma.

## 1. Introduction

Soft tissue sarcomas are often mistaken for benign tumors, and upwards of one in three tumors are initially excised without an appropriate biopsy or adequate margins [1]. The impact of prior unplanned excision on outcomes following oncologic resection is debated; however, re-excision is often performed for tumors which were previously excised in a non-oncologic fashion [2,3,4,5,6]. Among those who undergo re-excision, the presence of residual tumor has been consistently demonstrated to be associated with worse oncologic outcomes [7,8,9].

Synovial sarcomas in particular may be more prone to unplanned excision due to their tendency towards slow growth and juxta-articular location [10]. Even among patients who undergo planned excision and multidisciplinary therapy, five-year survival rates range from 59 to 75% [11,12,13,14,15]. The literature on outcomes following unplanned excision of synovial sarcoma is limited [5,9]. We therefore sought to review the factors associated with recurrence and survival following unplanned excision of synovial sarcoma at our institution.

## 2. Methods

Following Institutional Review Board approval, our institution’s diagnostic index database was queried for all patients with a histopathologic diagnosis of synovial sarcoma who were evaluated between 1997 and 2022. Patients presenting following treatment at another institution without records of their initial treatment were excluded. The medical records for all included patients were reviewed to collect data on demographics, tumor features, method of diagnosis, treatment course, and outcomes. Particular attention was paid to the manner in which patients were diagnosed with synovial sarcoma and whether diagnosis was obtained via unplanned excision. Unplanned excision was defined as surgical removal of the tumor prior to biopsy and without wide margins.

In total, 246 patients with synovial sarcoma were included for analysis. Eighty-seven patients (35%) were diagnosed after unplanned excision of their primary tumor (Table 1). Planned resection was performed in 144 patients (59%) and 13 patients (5%) were diagnosed via biopsy and did not undergo surgery. In the patients who underwent unplanned excision, the median follow-up in surviving patients was 60 months (range 1–235). Patients were included irrespective of their length of follow-up for the overall rate of unplanned excision and demographic information; patients lost to follow-up within 12 months were excluded from survival analysis.

Primary outcomes consisted of local recurrence-free survival (LRFS), metastasis-free survival (MFS), and disease-specific survival (DSS). Chronologic endpoints such as duration of follow-up, recurrence-free survival, and disease-specific survival were calculated from the initial date of diagnosis with synovial sarcoma. Categorical variables were reported as frequencies and percentages, while continuous variables were reported as mean or median values with ranges. The relationship between clinical or treatment features and survival were assessed using Cox Hazard Ratios with or without time-varying covariates. For the purposes of this study, a *p*-value < 0.05 was considered to be statistically significant. LRFS, MFS, and DSS were analyzed using the Kaplan–Meier method. Statistical analyses were performed via the BlueSky Statistics software package (Version 10.3.4).

## 3. Results

### 3.1. Unplanned vs. Planned Excision

Primary tumors which underwent unplanned excision tended to be smaller than those which underwent oncologic excision (mean maximum dimension 4.8 cm vs. 8.4 cm, *p* = 0.01), were more likely to be located in the extremity (OR 1.88, 95% CI [1.05, 3.33], *p* = 0.03), less likely to be located in the chest (OR 0.26, 95% CI [0.12, 0.59], *p* = 0.001), and less likely to present with metastatic disease (OR 0.25, 95% CI [0.07, 0.86], *p* = 0.04). Compared to tumors which were initially excised in an oncologic fashion, tumors which underwent unplanned excision had a similar LRFS (HR 0.78, 95% CI [0.43, 1.45], *p* = 0.4). Patients who underwent unplanned excision had an improved MFS (HR 0.45, 95% CI [0.28, 0.71], *p* < 0.001) and DSS (HR 0.53, 95% CI [0.34, 0.86], *p* = 0.01). However, there was no difference in MFS or DSS when controlling for tumor size (*p* > 0.10).

### 3.2. Treatment Following Unplanned Excision

Twelve patients did not undergo re-excision following unplanned resection. Eleven of these patients received definitive radiotherapy and five received chemotherapy. Of the remaining 75 patients, 61 underwent re-excision with negative margins, 11 underwent major limb amputation, and 3 underwent re-excision with positive margins. Re-excision occurred at a mean of 3.6 months from the initial unplanned procedure (range 0.5–8.5 months). 

Chemotherapy was administered following unplanned excision in 39 patients. This was administered prior to definitive surgery in 21 patients, following definitive surgery in 17 patients, and in lieu of surgery in 1 patient. Chemotherapy was given concurrent with radiation in 27 patients. The most common chemotherapy regimen was doxorubicin and ifosfamide (n = 28) followed by methotrexate, doxorubicin, and cisplatin (n = 7). Three patients developed metastatic disease in the interim between unplanned excision and re-resection, and all three received chemotherapy.

Sixty-three patients received radiotherapy. Radiation was delivered only prior to definitive surgery in 26 patients (mean dose 49 Gy, range 43–60), only following definitive surgery in 17 patients (mean dose 60 Gy, range 50–70), and in lieu of surgery in 5 patients (mean 59 Gy, range 45–70). Thirteen patients received intraoperative radiotherapy along with pre-operative (n = 11) or post-operative (n = 2) radiation, and three patients received brachytherapy.

### 3.3. Recurrence and Survival after Unplanned Excision

**Figure 1:**
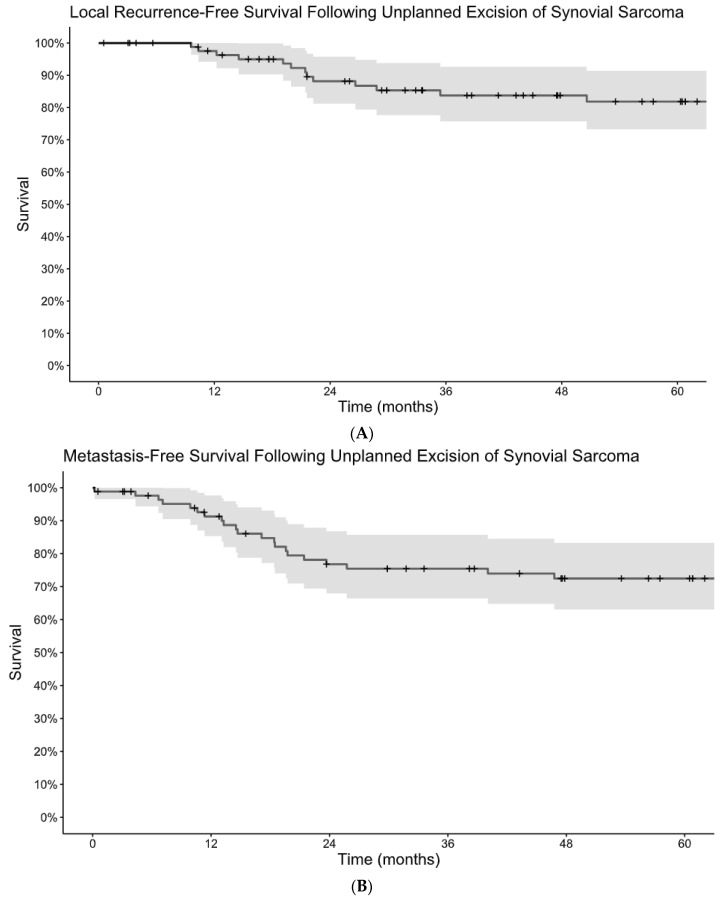
Kaplan–Meier estimates of local recurrence-free survival (**A**) and metastasis-free survival (**B**) following unplanned excision of synovial sarcoma.

**Figure 2:**
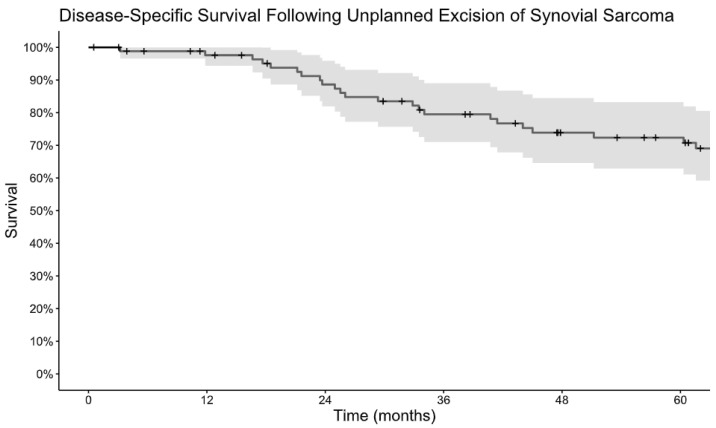
Kaplan–Meier estimate of disease-specific survival following unplanned excision of synovial sarcoma.

Among patients who underwent unplanned excision, LRFS was 98% at 1 year and 82% at 5 years and MFS was 91% at 1 year and 72% at 5 years. (Figure 1A,B). DSS was 98% at 1 year and 72% at 5 years (Figure 2). Monophasic subtype and axial location were risk factors for disease recurrence (Table 2). Re-excision was associated with a lower risk of local recurrence and disease-specific mortality. The absence of residual tumor on re-excision was protective against local recurrence, the development of metastatic disease, and disease-specific death. Patients who received radiation, including both neoadjuvant and adjuvant, had a lower risk of progression to metastatic disease and disease-specific death, but there was no significant association with local recurrence. There was no difference in terms of recurrence or survival for patients who received chemotherapy. 

**Figure 3:**
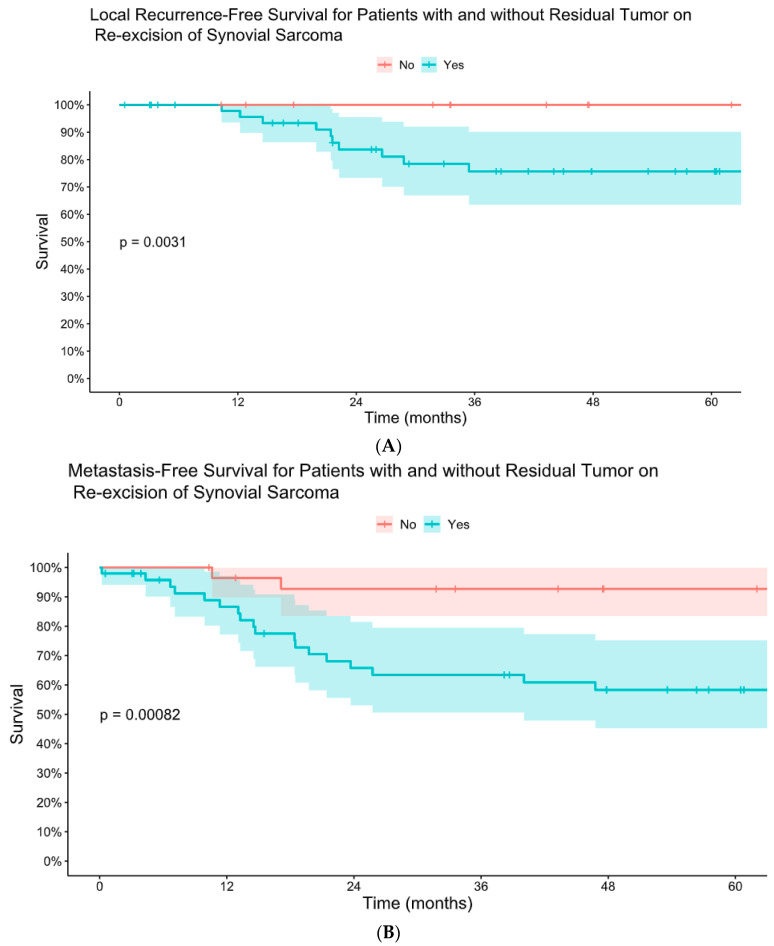
Kaplan–Meier comparative estimates of local recurrence-free survival (**A**) and metastasis-free survival (**B**) for patients with and without residual tumor on re-excision of synovial sarcoma after initial unplanned excision.

**Figure 4:**
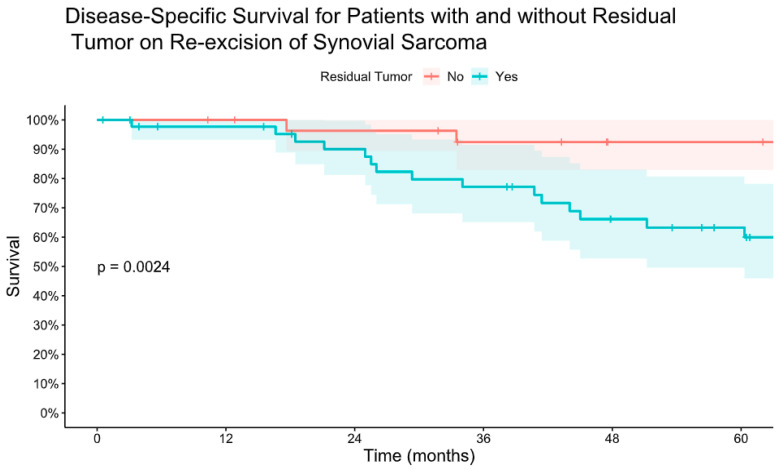
Kaplan–Meier estimate of disease-specific survival for patients with and without residual tumor on re-excision of synovial sarcoma after initial unplanned excision.

Forty-six patients (61%) had residual tumor on pathology analysis following re-excision. The presence of residual tumor on re-excision was associated with worse LRFS, MFS, and DSS (Figure 3 and Figure 4). There was no difference in mean time to re-excision between those with and without residual tumor (3.2 vs. 3.9 months, *p* = 0.24). There were no instances of local recurrence among the patients without residual tumor on re-excision (median follow-up 6.3 years, range 0.8–18). Five-year MFS was 93% for patients without residual tumor on re-excision and 58% for patients with residual tumor on re-excision. Five-year DSS was 92% for those without residual tumor and 63% for those with residual tumor. 

Patients who received pre-operative radiation were less likely to have residual tumor on re-excision (OR 0.08, 95% CI [0.03, 0.25], *p* < 0.001); however, the effect of residual tumor on metastasis-free and disease-specific survival remained present after controlling for the administration of radiotherapy (MFS: HR 6.4, 95% CI [1.42, 29.0], *p* = 0.02; DSS: HR 5.86, 95% CI [1.27, 26.9], *p* = 0.02). When controlling for the presence of residual tumor, the association between radiation and MFS and DSS was not significant (MFS: HR 0.54, 95% CI [0.23, 1.27], *p* = 0.16; DSS: HR 0.46, 95% CI [0.18, 1.13], *p* = 0.09). Subgroup analysis did not detect a significant impact of post-operative radiation on recurrence or survival for patients with residual tumor on re-excision (*p* > 0.30). Patients who underwent pre-operative radiotherapy had a longer time to re-excision than those who did not (4.0 vs. 3.2 months, *p* < 0.01). Local recurrence was associated with worse DSS (HR 8.64, 95% CI [3.73, 20.0], *p* < 0.001) on time-dependent covariate analysis.

## 4. Discussion

One-third of patients with soft tissue sarcoma undergo unplanned excision of their primary tumors [1]. However, few studies have specifically investigated the treatment and outcomes of synovial sarcoma after unplanned excision [5,9,16]. In this study, we present our institution’s experience with the treatment and outcomes of 87 patients who previously underwent unplanned excision of synovial sarcoma. We found that unplanned excision did not impact recurrence or survival; however, the use of radiotherapy and the absence of residual tumor on re-excision were positive prognostic factors in oncologic outcomes.

In this series, the rate of prior unplanned excision for patients with synovial sarcoma was 35%, consistent with previous systematic reviews [1,3]. While univariate analysis demonstrated lower rates of metastasis and disease-specific death among those who underwent unplanned excision, this relationship disappeared when controlling for tumor size as these masses tended to be smaller. Additional risk factors for recurrence included monophasic subtype and axial location of the tumor. We previously found that unplanned surgery was not associated with recurrence-free survival in a small cohort of patients with synovial sarcoma of the upper extremity [16]. On the contrary, Yuan et al. reported that synovial sarcomas which underwent unplanned excision and subsequent re-excision had worse recurrence-free survival, metastasis-free survival, and disease-specific survival compared to planned excisions; however, it is unclear whether they accounted for differences in tumor size between the two groups [9]. The true impact of unplanned excision on synovial sarcoma is therefore debatable; however, larger studies on all soft tissue sarcomas have consistently demonstrated that unplanned excision followed by negative margin re-excision leads to similar oncologic survival compared to those with an initial planned resection [3,7,17]. 

The majority of patients in this series underwent radiotherapy following unplanned excision, typically prior to re-excision. The decision to perform radiotherapy was often on the basis of larger tumor size or planned close margins. In this series, radiation was found to be protective against the development of metastatic disease and disease-specific death, though did not meet statistical significance for association with local recurrence. Large registry database studies on synovial sarcoma have previously demonstrated improved survival with radiotherapy [18,19]. Relative to these studies, our cohort demonstrated more marked effects of radiation therapy on recurrence and survival which may be due to the smaller size of tumors which were inadvertently excised. Radiation was most commonly delivered prior to re-resection in this study, and no difference in survival or recurrence was detected between pre-operative and post-operative radiotherapy. Patients who underwent pre-operative radiotherapy were less likely to have residual synovial sarcoma on re-excision, and the association between radiation and outcomes weakened when controlling for the presence of residual tumor. 

Over 80% of tumors in our series underwent re-resection or amputation following unplanned excision. While the Cox Hazard analysis reached statistical significance for re-excision as a protective factor for survival but not recurrence, there was a strong trend towards lower recurrence despite there being only 12 patients who did not undergo repeat surgery. Among tumors which were re-excised, the presence of residual tumor was a highly unfavorable prognostic indicator. Yuan et al. demonstrated similar findings in their study on synovial sarcoma [9], though Zhang et al. found no difference in survival between those with and without residual tumor on re-excision of synovial sarcoma [5]. Other studies on soft tissue sarcomas, though not specific to synovial sarcoma, do support our findings that residual disease on re-excision is a risk factor for recurrence and disease-specific mortality [8,20,21,22,23]. However, most retrospective studies on unplanned excision are confounded by the fact that these tumors typically receive more aggressive treatment. Additionally, as knowledge of sarcoma genomics increases, the role of genetic markers as prognostic factors for certain sarcoma subtypes is becoming more clear [24,25,26]. While we did not evaluate the role of different SS18-SSX fusions in our series, it remains a possibility that the presence of certain genetic rearrangements underlies the findings in this study.

This study has several limitations. All data was retrospectively obtained from encounters at a single academic institution over a twenty-five-year period and therefore may not be generalizable to other regions or centers. The sample size is small due to the rarity of synovial sarcoma tumors, a minority of which are subjected to unplanned excision. This limits the ability to perform in-depth multivariate analysis and detect smaller effect sizes. Additionally, variations in treatment strategies such as chemotherapy regimen, radiation dosage and timing, and width of surgical margins contribute to the heterogeneity of the data and may confound our analysis. Multi-institutional collaborative studies on unplanned excision of synovial sarcoma may be needed to generate sufficient sample sizes to detect more subtle associations between risk factors and outcomes in this population.

## 5. Conclusions

One-third of patients presenting with synovial sarcoma were diagnosed after unplanned, non-oncologic excision. Patients with large, axial tumors had worse survival. Approximately half of patients who underwent unplanned excision had no residual tumor after pre-operative radiation. The use of radiation was associated with improved metastasis-free and disease-specific survival. Our results suggest that margin-negative re-resection and radiotherapy should be considered when feasible following unplanned excision of synovial sarcoma.

## Figures and Tables

**Table 1:** Baseline Characteristics of Patients Undergoing Excision of Synovial Sarcoma

	Unplanned Excision (n = 87)	Planned Excision (n = 144)
Age at diagnosis, mean (range), years	39 (5–85)	40 (5–81)
Females, proportion	56%	44%
Presenting tumor location, *n*		
Extremity	63	84
Abdomen	6	12
Chest/Intrathoracic	7	40
Head/Neck	8	6
Paraspinal	3	2
Bone involvement, proportion	5.7%	10%
Intraarticular involvement, proportion	9.0%	7.60%
Monophasic subtype, proportion	67%	67%
Tumor maximum dimension, mean (range), cm	4.8 (0.9–15)	8.4 (1.7–37)
Maximum dimension < 5 cm, proportion	65%	28%
Distant metastases on presentation, *n* (%)	3 (3.4%)	18 (13%)

**Table 2:** Cox Hazard analysis for disease recurrence and disease-specific mortality.

	A. Local Recurrence		B. Development of Metastasis		C. Disease-Specific Mortality	
Risk Factor	Hazard Ratio (95% CI)	*p*-Value	Hazard Ratio (95% CI)	*p*-Value	Hazard Ratio (95% CI)	*p*-Value
Age > 55 years	1.39 (0.45, 4.31)	0.57	0.86 (0.29, 2.52)	0.78	1.10 (0.41, 2.94)	0.86
Male Gender	0.39 (0.13, 1.21)	0.10	0.83 (0.36, 1.91)	0.66	0.84 (0.37, 1.88)	0.66
Size < 5 cm	0.88 (0.30, 2.58)	0.82	0.25 (0.10, 0.60)	<0.01 *	0.15 (0.06, 0.39)	<0.001 *
Monophasic Subtype	3.10 (1.18, 8.19)	0.02 *	4.45 (1.01, 19.6)	0.04 *	4.11 (0.92, 18.2)	0.06
Lesion Location:						
Extremity	Reference	-	Reference	-	Reference	-
Abdomen	7.22 (1.77, 29.4)	0.01 *	1.76 (0.39, 7.90)	0.46	2.22 (1.19, 16.0)	0.03 *
Chest	10.2 (2.73, 38.0)	0.001 *	3.68 (1.18, 11.5)	0.03 *	3.33 (2.15, 19.1)	<0.001 *
Head/Neck	2.85 (0.59, 13.8)	0.19	2.43 (0.68, 8.64)	0.17	2.10 (1.00, 11.2)	0.06
Paraspinal	1.64 (0.86, 12.5)	0.14	6.24 (1.38, 28.3)	0.02 *	2.59 (1.64, 35.0)	0.01*
Treatment Included:						
Re-Excision	0.14 (0.05, 0.38)	<0.001 *	0.67 (0.23, 1.98)	0.47	0.35 (0.14, 0.88)	0.03 *
Radiation Therapy	0.61 (0.21, 1.79)	0.37	0.31 (0.13, 0.70)	<0.01 *	0.35 (0.15, 0.79)	0.01 *
Chemotherapy	1.35 (0.51, 3.60)	0.55	2.07 (0.90, 4.80)	0.90	1.73 (0.96, 3.11)	0.07
Residual Tumor on Re-excision	NA ^ⱡ^	-	8.17 (1.90, 35.2)	<0.01 *	7.49 (1.67, 33.6)	0.01 *

NA ^ⱡ^: Not applicable, no local recurrence events in the group without residual tumor on re-excision * *p* < 0.05.

## Data Availability

The data presented in this study are available on request from the corresponding author.
